# Surgical Complication Following Urgent Appendectomy and Considerations in Surgical Management of a Patient With Mitochondrial Encephalopathy, Lactic Acidosis, and Stroke-Like Episodes (MELAS)

**DOI:** 10.7759/cureus.39129

**Published:** 2023-05-17

**Authors:** Nicholas Cassimatis, Francis Ruzicka, Kaitlin Reilly, Rajat Kumar

**Affiliations:** 1 Neurological Surgery, Hackensack Meridian School of Medicine, Hackensack, USA; 2 Neurology, Hackensack Meridian School of Medicine, Hackensack, USA; 3 Neurology-Neurological Critical Care, Hackensack University Medical Center, Hackensack, USA; 4 Neurology-Stroke, Hackensack University Medical Center, Hackensack, USA

**Keywords:** surgical management, general surgery, complication, appendectomy, surgery, neurology, stroke, lactic acidosis, mitochondrial encephalopathy, melas

## Abstract

This case report details a surgical complication with a delayed presentation in a 23-year-old male with mitochondrial encephalopathy, lactic acidosis, and stroke-like episodes (MELAS). MELAS is a rare entity that can complicate the routine medical and surgical management of patients. Without sufficient research and guidelines, decision-making for patients who require time-sensitive care may be difficult. This patient population may require special consideration and preventative measures to maximize safety in their surgical care. This case serves to highlight a surgical complication that MELAS patients may be susceptible to, as well as to detail possible means of prevention and protection.

## Introduction

Mitochondrial encephalopathy, lactic acidosis, and stroke-like episodes (MELAS) are a syndrome characterized by mutations in the deoxyribonucleic acid (DNA) in maternal mitochondrial DNA and nuclear DNA [1]. The result of these multi-organ dysfunctions with symptoms that often include but are not limited to mitochondrial myopathy, encephalopathy, stroke-like episodes, seizures, lactic acidosis, recurrent headaches, and renal impairment [1-4]. MELAS is a relatively uncommon entity, affecting fewer than one in 4000 individuals, and usually presents in childhood or early adulthood, though late presentations have been described in the literature [4,5]. MELAS can make medical and surgical management of patients substantially more difficult, especially with no clear guidelines on caring for this unique patient population. In this report, we present the case of a 23-year-old male patient with a known diagnosis of MELAS, whose condition complicated his surgical management.

## Case presentation

HPI

A 23-year-old male with a past medical history of SOX5 gene mutation, intellectual disability, epilepsy, psychogenic nonepileptic seizures (PNES), and recently diagnosed MELAS presented to the emergency room with episodes of loss of consciousness and headache. The patient reported having dizziness before losing consciousness, and this occurred multiple times a day. The headaches were described as sharp and occurring on his forehead. He endorsed additional symptoms of left upper and lower extremity pain, as well as chest pain, but denied photophobia, phonophobia, nausea, or vomiting. The patient's family reported mood changes and weight loss but denied staring spells, fecal or urinary incontinence, and generalized tonic-clonic activity. They confirmed his independence with activities of daily living.

Vitals/PE

On admission, the patient’s vitals were stable. He was awake and alert, but he answered questions and followed commands inconsistently. Orientation was difficult to gauge due to the patient's clinical condition. Cranial nerves, sensation to light touch, and coordination were all intact bilaterally; however, strength was 4/5 on the Medical Research Council (MRC) Scale for muscle strength in the left upper extremity, and both bulk and tone were decreased throughout. Labs were ordered, neurology was consulted, and the patient was sent for head computed tomography (CT) and video electroencephalogram (vEEG) monitoring.

Imaging/Labs

**Figure 1:**
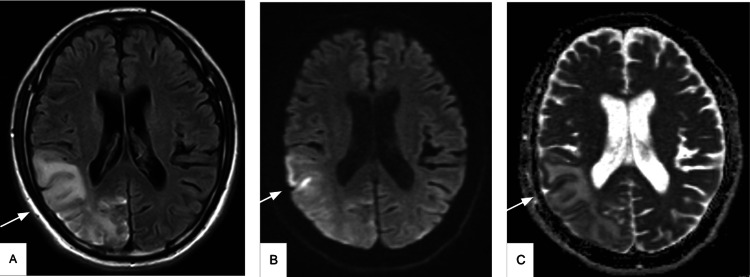
Preoperative MRI brain Gyriform cortical diffusion restriction involving the right temporoparietal and occipital regions but sparing the subjacent subcortical white matter (white arrows), suggesting tissue injury in a nonvascular distribution. A. T2-weighted fluid-attenuated inversion recovery (FLAIR). B. Diffusion-weighted imaging (DWI). C. Apparent diffusion coefficient (ADC).

A complete blood count, a complete metabolic panel, coagulation, and urinalysis all returned within normal limits. Total creatinine kinase was elevated at 280 [iU]/L. Lactic acid was elevated at 2.7 mmol/L. Head CT revealed an age-indeterminate infarct involving the right parietal, occipital, and a portion of the right temporal lobe, so further evaluation with magnetic resonance imaging (MRI) was recommended. MRI revealed an infarct that was at least two weeks old and confirmed findings compatible with MELAS: gyriform cortical diffusion restriction involving the right temporoparietal and occipital regions but sparing the subjacent subcortical white matter, suggesting tissue injury in a nonvascular distribution (Figure 1). vEEG showed diffuse slowing and right temporal slowing consistent with a right temporal structural or functional problem.

The patient was continued on his home medications of clobazam, gabapentin, levetiracetam, phenobarbital, levocarnitine, vitamin D, and folic acid. He was started on vitamin E, coenzyme Q10, and arginine. During admission and laboratory work-up, the patient’s neurologic status remained stable.

Surgery

**Figure 2:**
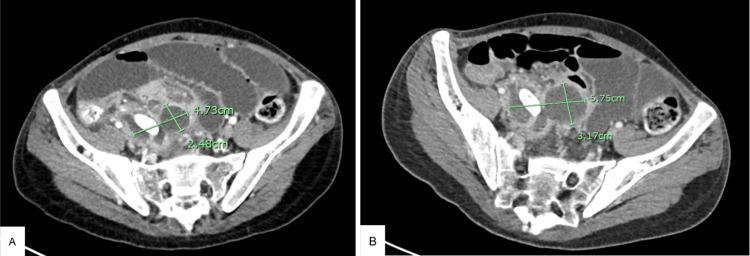
Preoperative CT abdomen and pelvis 2A. Initial imaging revealed perforated appendicitis with a small abscess and small bowel obstruction. (measurements in green) 2B. Repeat imaging revealed perforated appendicitis with a slight interval increase in size of a bilobed periappendiceal abscess. Persistent small bowel obstruction. (measurements in green)

Roughly one week after admission, the patient began complaining of abdominal pain and was noted to have a low-grade fever and increased white blood cells. A CT of the abdomen and pelvis (A/P) was ordered and revealed a perforated appendicitis with a small abscess and small bowel obstruction (Figure 2A). Acute surgical intervention was deemed unnecessary, and IV piperacillin-tazobactam was started with the intention to follow up with an outpatient interval appendectomy. After five days, CT A/P was repeated and revealed a slight increase in the size of a bilobed periappendiceal abscess (Figure 2B). At this point, the decision was made to take the patient to surgery. A laparoscopic robot-assisted appendectomy was performed with washout and drainage of the appendiceal abscess. Enterotomy repair was also performed due to small bowel perforation during abscess removal; however, there were no apparent postoperative complications at that time.

Complications/Discharge

**Figure 3:**
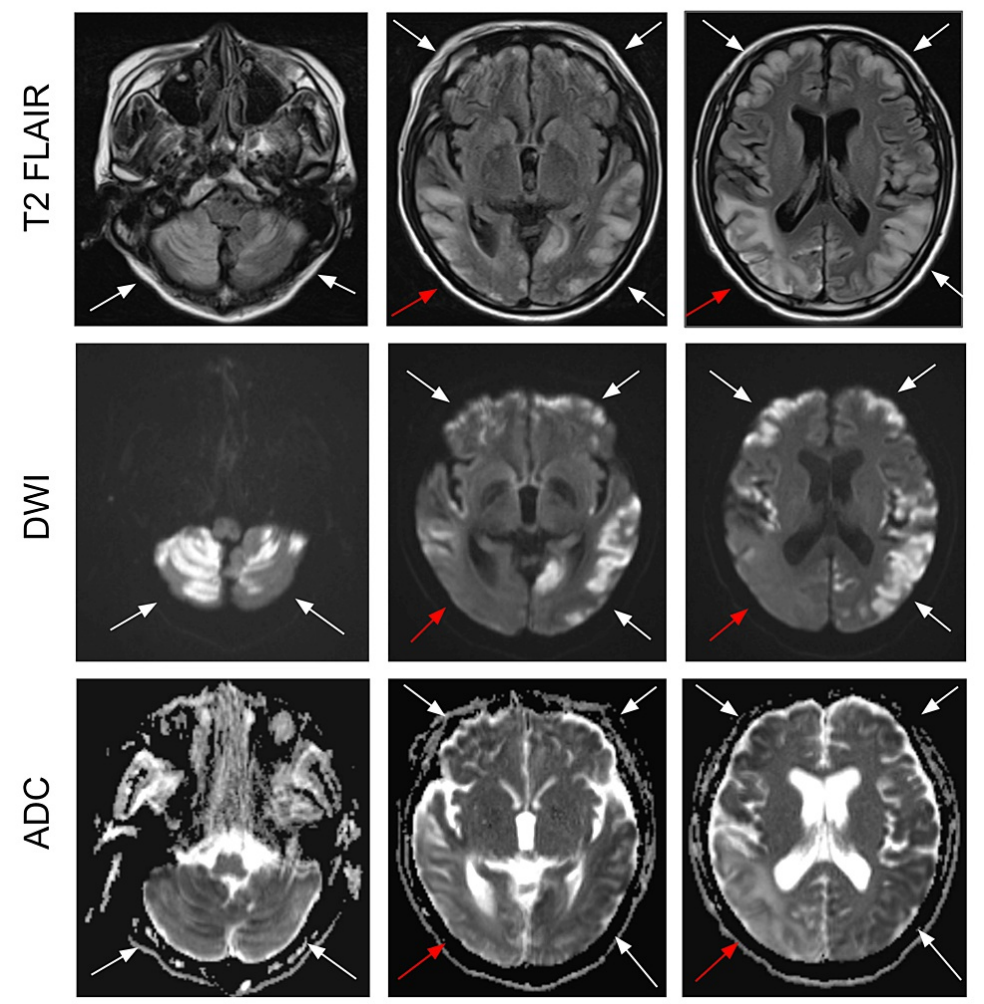
Postoperative MRI brain Significant worsening with progression of bilateral supra and infratentorial variables extensive increased FLAIR and T2-weighted with high diffusion and low ADC signal. Findings are most consistent with ischemic changes with multiple bilateral new (white arrows) and previous right parietal (red arrow) infarctions.

On a post-operative day one, neurology was re-consulted to evaluate the new-onset right-sided visual loss and recommended imaging as well as the discontinuation of all enzyme-inducing anti-epileptic medications, particularly phenobarbital, which the patient was taking at the time. Over the next two postoperative days, the patient’s clinical status deteriorated, demonstrating acute hypoxic respiratory failure warranting a rapid response, worsening ischemic changes with multiple bilateral infarcts revealed on imaging (Figure 3), worsening mental status warranting a second rapid response, and aspiration pneumonia, eventually resulting in intubation and transfer to the surgical intensive care unit (SICU). In the SICU, the patient was unable to be extubated, so the decision was made to place a tracheostomy tube. A percutaneous endoscopic gastrostomy (PEG) tube was placed at this time as well. The patient remained stable on the tracheostomy collar and was ultimately downgraded to medical floors for further management. He was discharged to subacute rehab after a 67-day hospital stay.

## Discussion

This case provides a valuable example and learning tool in terms of highlighting the difficulties in the surgical care of MELAS patients. The case presented in this report chronicles the presentation and surgical care of a patient with MELAS and a subsequent postoperative complication. While the patient’s surgery is generally considered a routine surgical issue, the patient’s MELAS made some choices more difficult, and there are many aspects of the care that could be learned from and improved upon. The MELAS population is unlike the general population in that they may be very sensitive to oxidative stress, and much of their care must be planned through this lens. 

Initially, the patient was treated conservatively and not taken to surgery for his appendicitis. The aim of this measure was again to reduce the oxidative stress that would follow surgery. However, once it was discovered that the patient had perforated appendicitis with a periappendiceal abscess and small bowel obstruction, it may have been more prudent to take the patient for surgery urgently, as this condition resulted in dramatic stress for the patient. Some considerations that could have minimized stress during surgery are pre-treatment with IV L-Arginine, L-Citrulline, or another agent protective against oxidative stress [6]. Although there were no known episodes of intraoperative hypotension in this case, maintaining appropriate blood pressure is another important precaution for minimizing stress. Furthermore, the patient’s phenobarbital was not discontinued, increasing the patient’s susceptibility to aspiration. As detailed in the case section, two days following the patient’s appendectomy and abscess drainage, the patient was found to have a new ischemic stroke and new aspiration pneumonia. 

This case highlights the importance of surgical care considerations in the context of MELAS patients. Interdisciplinary care with frequent communication between neurological, genetic, and surgical specialties is also highly important and may have helped prevent perioperative stroke risk. For patients with MELAS, it is crucial to have interdisciplinary conversations to try to reduce perioperative stroke risk and to anticipate complications that may arise in the context of specific patients. Unfortunately, there are no unifying guidelines on the management of MELAS, especially in the context of surgical care [7,8]. This further complicates management and highlights the need for conversations around this topic. This case serves to call attention to the need for special considerations, planning, and management in the surgical care of patients with MELAS. 

## Conclusions

MELAS is a rare syndrome that currently lacks unifying guidelines to help direct its management. Because of the altered mitochondrial metabolism that accompanies MELAS, patients with it are more susceptible to damage from stress, such as surgery in the case of this patient. Even for routine surgical procedures, special considerations and preparations should be made to reduce the burden placed on their bodies. To demonstrate, we present this case of a 23-year-old male patient with a known diagnosis of MELAS whose condition complicated surgical management of perforated appendicitis with an appendiceal abscess, resulting in worsened neurological status, aspiration pneumonia, and subsequent prolonged intensive care. Ultimately, more research must be done to improve interdisciplinary management and reduce complications in the surgical care of patients with MELAS.
